# Inclusion of a degron reduces levelsof undesired inteins after AAV-mediated protein*trans-*splicing in the retina

**DOI:** 10.1016/j.omtm.2021.10.004

**Published:** 2021-10-19

**Authors:** Patrizia Tornabene, Ivana Trapani, Miriam Centrulo, Elena Marrocco, Renato Minopoli, Mariangela Lupo, Carolina Iodice, Carlo Gesualdo, Francesca Simonelli, Enrico M. Surace, Alberto Auricchio

**Affiliations:** 1 Telethon Institute of Genetics and Medicine (TIGEM), Pozzuoli 80078, Italy; 2 Medical Genetics, Department of Translational Medicine, Federico II University, Naples 80131, Italy; 3 Eye Clinic, Multidisciplinary Department of Medical, Surgical and Dental Sciences, University of Campania L. Vanvitelli, Naples 80131, Italy; 4 Medical Genetics, Department of Advanced Biomedicine, Federico II University, Naples 80131, Italy

**Keywords:** protein *trans*-splicing, split-inteins, intein degradation, ecDHFR, gene therapy, AAV, Stargardt disease (STGD1), inherited retinal disease

## Abstract

Split intein-mediated protein *trans-*splicing expands AAV transfer capacity, thus overcoming the limited AAV cargo. However, non-mammalian inteins persist as *trans-*splicing by-products, and this could raise safety concerns for AAV intein clinical applications. In this study, we tested the ability of several degrons to selectively decrease levels of inteins after protein *trans-*splicing and found that a version of *E. coli* dihydrofolate reductase, which we have shortened to better fit into the AAV vector, is the most effective. We show that subretinal administration of AAV intein armed with this short degron is both safe and effective in a mouse model of Stargardt disease (STGD1), which is the most common form of inherited macular degeneration in humans. This supports the use of optimized AAV intein for gene therapy of both STGD1 and other conditions that require transfer of large genes.

## Graphical abstract

**Figure:**
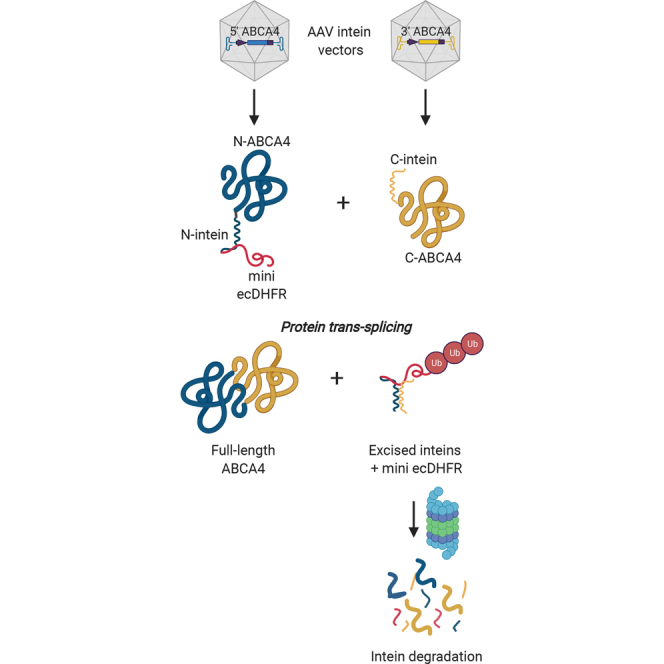

## Teaser

Intein-mediated protein *trans*-splicing (PTS) expands AAV gene transfer capacity. However, non-mammalian inteins persist as *trans*-splicing by-products. Here, we show that ecDHFR selectively degrades inteins after PTS and that AAV intein vectors armed with this degron are both safe and effective in the retina of a mouse model of genetic blindness.

## Introduction

Retinal gene therapy is emerging as the leading approach for the treatment of different forms of inherited blindness.^1^ Most of the ongoing clinical trials are based on adeno-associated viral (AAV) vectors and show low immunogenicity and long-term transgene expression after a single administration.^2^, ^3^, ^4^ The most successful example of this is Luxturna, the first approved gene therapy for an ocular disease^5^ based on AAV.

However, the relatively small DNA packaging capacity of AAV, which is restricted to the size of the parental genome (≈5 kb^6^) prevents their application for the treatment of diseases that arise due to mutations in genes with larger coding sequences. In past years, significant efforts have been made to overcome the limited transfer capacity of AAV vectors.^7^ Recently, intein-mediated protein *trans-*splicing (PTS) has been evaluated as a strategy to reconstitute large proteins via AAV vectors, thus overcoming their limited cargo capacity.^8^, ^9^, ^10^ In this system, a large protein coding sequence is split into two or more parts each flanked by sequences that encode split-inteins, which are independently cloned in two AAV vectors. Split-inteins are expressed as two independent polypeptides (N-intein and C-intein) at the extremities of the host polypeptides (N-polypeptide and C-polypeptide) and remain inactive until encountering their complementary partner.^11^ On counterpart association, the reconstituted intein precisely excises itself from the host protein while mediating ligation of the N- and C-polypeptides via a peptide bond, in a traceless manner.

We have recently shown that the AAV intein system reconstitutes large therapeutic protein in the retina of animal models and in human photoreceptors from retinal organoids.^9^ We showed efficient reconstitution of the large ATP-binding cassette subfamily A member 4 (ABCA4) protein, which encodes the photoreceptor (PR)-specific *all-trans*-retinal transporter.^12^ ABCA4 is critically important for the clearance of photoisomerized *all*-*trans*-retinal from the photoreceptor disk lumen. Non-functional ABCA4 leads to accumulation of lipofuscin pigments in the retinal pigment epithelial (RPE) cells, triggering RPE-cell death and subsequent PR degeneration causing vision loss in patients with Stargardt disease (STGD1, OMIM: #248200), which is the most common inherited macular dystrophy in humans. We also showed that AAV-ABCA4 intein vectors improve the retinal phenotype of a mouse model of STGD1.^9^ While effective in the retina, AAV intein vectors encode components (excised inteins) of non-mammalian origin that could elicit immune or toxic responses in target cells and/or raise regulatory concerns for clinical translation.

**Figure 1:**
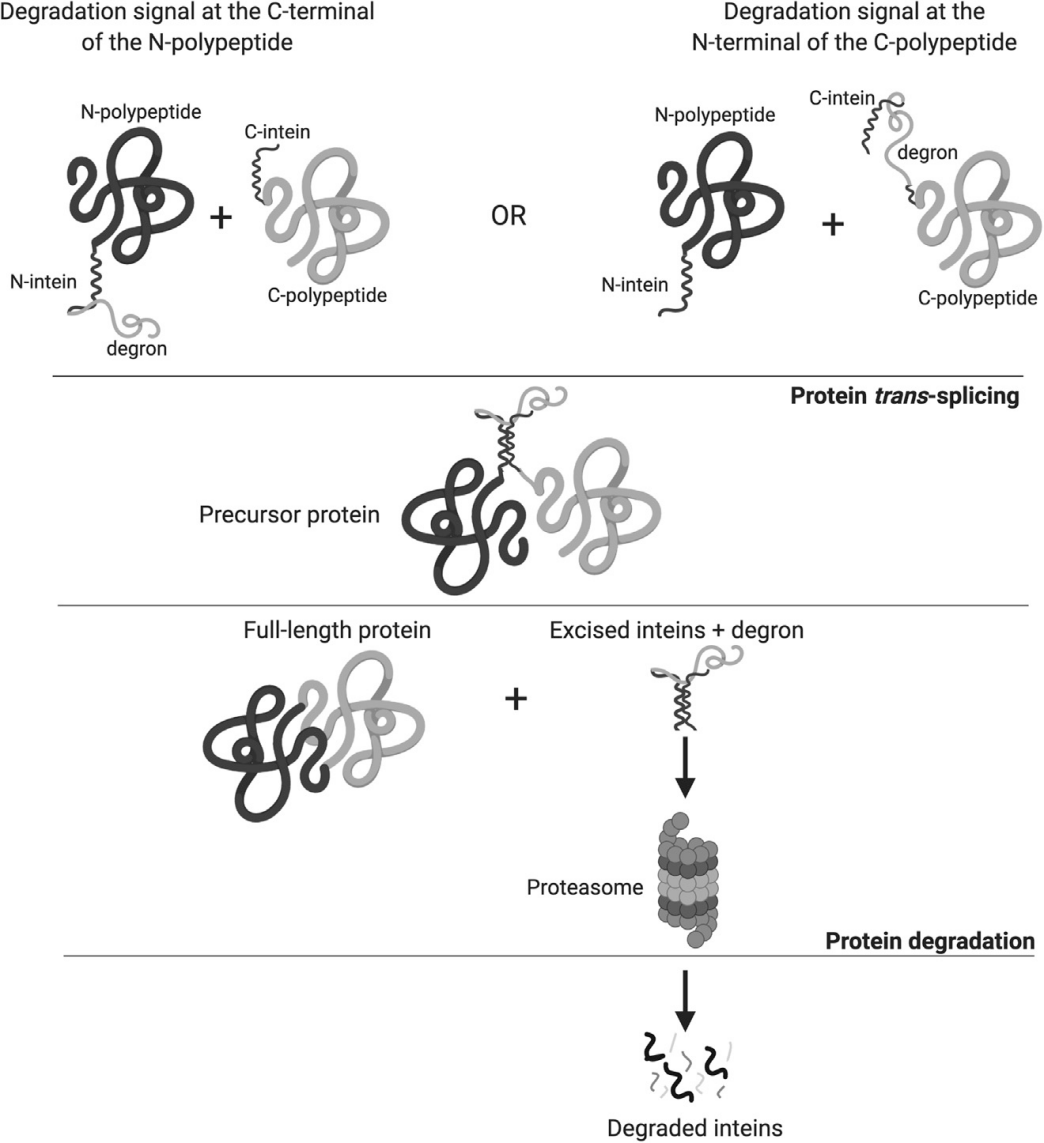
Schematic representation of selective intein degradation following protein *trans-*splicing The coding sequence of a large protein is split into halves (N- and C-polypeptides), flanked by the split-inteins (N- and C-inteins), which are fused to a degradation signal (degron) at either the N- or C-terminus of the intein. Pairing of two-half polypeptides is mediated via intein self-recognition; subsequent intein self-excision from the host protein results in full-length protein reconstitution. The degron, now embedded within the excised inteins, is rapidly ubiquitinated and degraded by the proteasome.

To prevent this potential issue, we have evaluated the inclusion of a degron in the *trans*-splicing system, which, once embedded within the excised inteins, mediates their rapid ubiquitination and subsequent proteasomal degradation (Figure 1).

Signals that mediate or are predicted to mediate ubiquitin-dependent proteasomal protein degradation were found in the (1) class II transactivator (CIITA^13^); (2) murine ornithine decarboxylase (ODC^14^); (3) short peptide CL1^15^; (4) survival of motor neurons (SMN2^16^); (5) *E. coli* dihydrofolate reductase (ecDHFR^17^).

We explored the ability of each signal to selectively degrade excised split-inteins without interfering with the *trans*-spliced full-length transgene products. In addition, we evaluated both the efficacy and safety of AAV intein with degron in the mouse retina in view of future clinical translations.

## Results

### Inclusion of the ecDHFR degron in the N-intein results in selective degradation of the excised inteins

To selectively reduce the amounts of the excised split-inteins after PTS has occurred in cells co-transduced with AAV intein vectors, several degradation signals have been included in the ABCA4 intein vectors (set 1 described in Tornabene et al.^9^) which include the *Nostoc punctiforme* DnaE split-inteins.

Each degradation signal was integrated at either the N- or C-terminal extremities of the split-inteins. The N- or C-terminal, or internal locations of the degradation signal with respect to the host polypeptide were chosen according to each signal's degradation activity preference. In addition, we ensured preservation of the internal His, the dipeptide His-Asn at the intein C terminus and the Cys at the beginning of both the N-intein and C-polypeptide which are all required for PTS to occur.^11^

We cloned (1) the CIITA signal at the 5′ end of the N-intein; (2) the CL1, SMN2, and ODC7 signals at the 3′ end of the C-intein; and (3) the ecDHFR at the 3′ end of the N-intein so that, upon PTS and intein excision, the degradation signals would be placed at either the N- (1) or C-terminals (2) or in the middle (3) of the excised inteins, respectively (see Figure S1 and Table 1).

**Table 1:** Degradation signals tested in this study

Degradation signal	Amino acid sequence	Terminal cloning position
CIITA	RPGSTSPFAPSATDLPSMPEPALTSR	N-DnaE (+4)
CL1	ACKNWFSSLSHFVIHL	C-DnaE (−1)
SMN	YMSGYHTGYYMEMLA	C-DnaE (−1)
ODC7	MSCAQES	C-DnaE (+16)
ecDHFR	ISLIAALAVDYVIGMENAMPWNLPADLAW FKRNTLNKPVIMGRHTWESIGRPLPGRKNII LSSQPSTDDRVTWVKSVDEAIAACGDVPEI MVIGGGRVIEQFLPKAQKLYLTHIDAEVEG DTHFPDYEPDDWESVFSEFHDADAQNSHS YCFEILERR	N-DnaE (end)

Then, we tested whether the fusion of the degradation signals with the inteins results in selective intein degradation *in vitro*.

To do this, HEK293 cells were co-transfected with the ABCA4 intein plasmids which included or not (control) the degradation signals. Cells were harvested 72 h post-transfection and cell lysates were analyzed by western blot with anti-3xflag antibodies.

**Figure 2:**
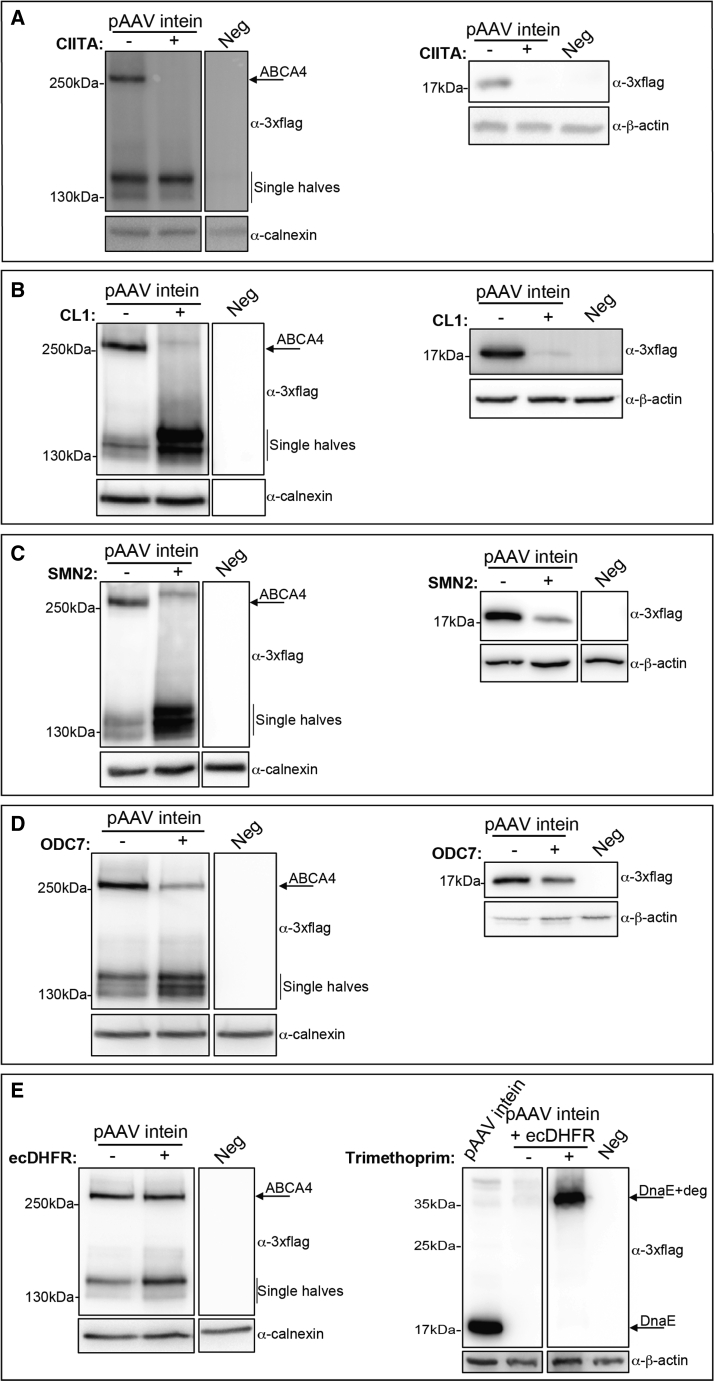
*E. coli* DHFR mediates selective intein degradation without affecting full-length ABCA4 expression Western blot (WB) analysis of lysates from HEK293 cells transfected with AAV intein plasmids including (+) or not including (−) the (A) CIITA, (B) CL1, (C) SMN, (D) ODC7, and (E) ecDHFR degrons. The arrows indicate the bands corresponding to full-length ABCA4 protein (ABCA4) (left panel) while excised inteins are shown in the right panel. WB analysis of lysates from HEK293 cells transfected with AAV intein-ecDHFR plasmids containing (+) or not containing (−) the stabilizer TMP. The arrows indicate the bands corresponding to excised inteins which include (DnaE + deg) or do not include (DnaE) the ecDHFR degron. Neg: untransfected cells. The WB is representative of N = 3 independent experiments.

We found no full-length ABCA4 expression from plasmids, including CIITA (Figure 2A left), despite successful expression of the predicted half polypeptides, suggesting that the inclusion of the degradation signal prevents proper recognition of the split-inteins. Accordingly, no excised inteins were observed (Figure 2A, right). Degradation signals at the 3′ end of the C-intein such as CL1, which we have previously tested successfully in the context of dual AAV vectors,^18^ interfere with PTS (Figure 2B). The presence of the untranspliced protein was more evident when using the C-terminal SMN2 degron (Figure 2C).

We also tested the ODC-derived degradation signal that is reported to be active even when located far from the C terminus. ODC7^14^ was included at position −17 of the C-intein, far from the aminoacidic region involved in the splicing. The inclusion of ODC7 negatively impacted on PTS efficiency but did not result in reductions to the levels of excised inteins (Figure 2D). The apparent low level of excised inteins is the direct consequence of the low amount of reconstituted full-length ABCA4. Overall, these data suggest that the inclusion of a degradation signal within either the N- or C-intein is detrimental to PTS efficiency (Figures 2A–2D).

We then evaluated the mutated form of the *E. coli* dihydrofolate reductase (ecDHFR^17^), a degradation signal that is active at internal positions. Despite being reported to be active also when placed at the C-terminal end of a protein^17^, ecDHFR is mostly used as an N-terminal destabilization domain.^19^, ^20^, ^21^ We, therefore, investigated ecDHFR ability to degrade inteins after PTS. Interestingly, the degradation activity of ecDHFR is inhibited by its small stabilizing ligand, trimethoprim (TMP). Therefore, in the presence of TMP, fusion proteins containing ecDHFR escape from proteasomal degradation.^17^ Notably, as shown in Figure 2E (right), we found that the inclusion of the ecDHFR degradation signal at the 3′ end of the N-intein strongly reduces the amounts of the excised inteins following PTS. TMP treatment inhibits this reduction, demonstrating that intein degradation is specifically due to ecDHFR activity. Importantly, the inclusion of the degradation signal did not interfere with PTS, thus resulting in similar amounts of full-length ABCA4 protein compared with cells co-transfected with ABCA4 intein plasmids without the degradation signal (Figure 2E, left). To confirm ecDHFR-mediated degradation is independent of the transgene product, we included ecDHFR in EGFP-intein plasmids (described in Tornabene et al.^9^) and found robust full-length EGFP expression with no detectable intein levels (Figure S2).

### A shortened version of ecDHFR induces the selective degradation of the excised inteins

**Figure 3:**
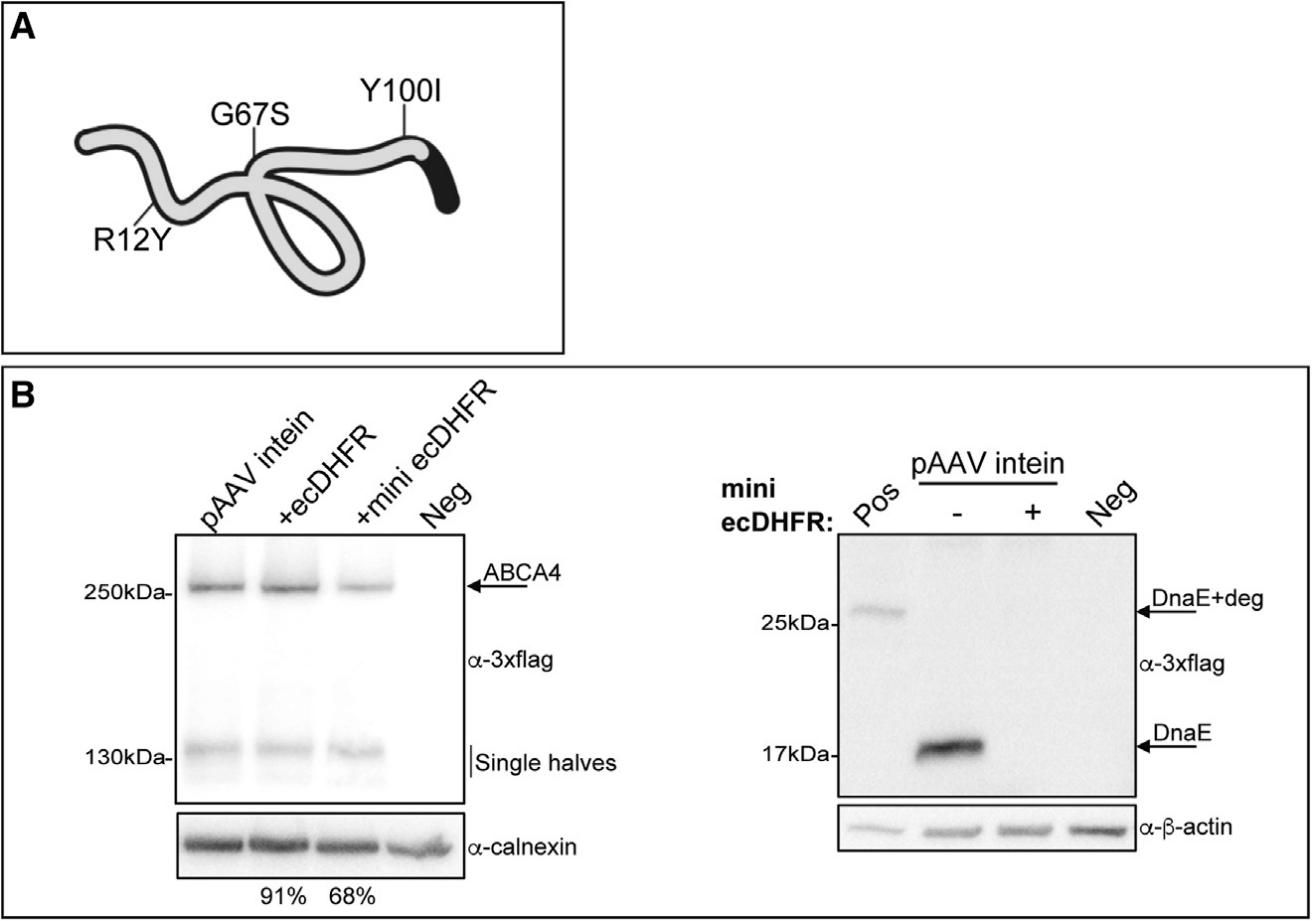
A shortened version of the *E. coli* DHFR retains its degradation activity in cells transfected with ABCA4 intein plasmids (A) Graphical representation of the mini ecDHFR (gray line) with the R12Y, G67S, and Y100I amino acid changes originally reported to be associated with protein degradation activity when ecDHFR (black line) was placed at the N terminus. (B) Western blot (WB) analysis of lysates from HEK293 cells transfected with AAV intein plasmids including or not including either the ecDHFR or mini ecDHFR degron. The arrows indicate bands corresponding to the full-length ABCA4 protein (ABCA4, left panel) while excised inteins (DnaE) are shown in the right panel. Pos: cell lysate of HEK293 cells transfected with ABCA4 intein-mini ecDHFR plasmids and treated with a proteasome inhibitor (MG132) to inhibit protein degradation since mini ecDHFR has lost trimethoprim sensitivity; Neg: untransfected cells. The percentage of both pAAV intein + ecDHFR and pAAV intein + mini ecDHFR ABCA4 expression relative to pAAV intein is depicted below the lanes. The Kruskal-Wallis test followed by Dunn's test was used to identify a statistically significant difference between groups; p = 0.092. The WB and quantification are representative of N = 3 independent experiments.

Although ecDHFR inclusion results in selective degradation of the excised inteins, its 477-nucleotide length can limit its application to AAV-mediated large gene transfer, given the small cargo capacity of AAV vectors (≈5 kb^6^). Therefore, we investigated whether reducing ecDHFR size could still result in intein degradation. We generated a “mini” ecDHFR that lacks the last 54 amino acids (which include those involved in sensitivity to TMP [Figure S3]) while retaining those reported to be crucial for its degradation activity (Figure 3A^17^). We found that inclusion of the mini ecDHFR at the C-terminal end of the N-intein results in intein degradation while preserving most full-length ABCA4 expression (Figure 3B, around 70% compared with about 90% obtained with ecDHFR).

Similar to the ABCA4 observations, the amount of full-length EGFP reconstituted in cells co-transfected with intein vectors including mini ecDHFR is similar to control cells while the inclusion of the degron results in almost undetectable intein levels (Figure S4).

### Inclusion of mini ecDHFR results in selective reduction of the excised intein in the mouse retina

We subretinally injected 4-week-old wild-type mice with AAV8 vectors that encode ABCA4 (with or without fused mini ecDHFR) under the control of the photoreceptor-specific human G protein-coupled receptor kinase 1 (GRK1) promoter. Animals were killed 4 weeks postinjection and protein expression in retinal lysates was evaluated by western blot. Full-length proteins were detected at variable levels, in seven of nine AAV-ABCA4 intein injected eyes (both including or not the mini ecDHFR), while the excised inteins were detected only in eyes injected with AAV-ABCA4 intein without the degradation signal (Figure S5).

### Increasing the amount of the N-ABCA4 half-polypeptide improves full-length protein reconstitution

**Figure 4:**
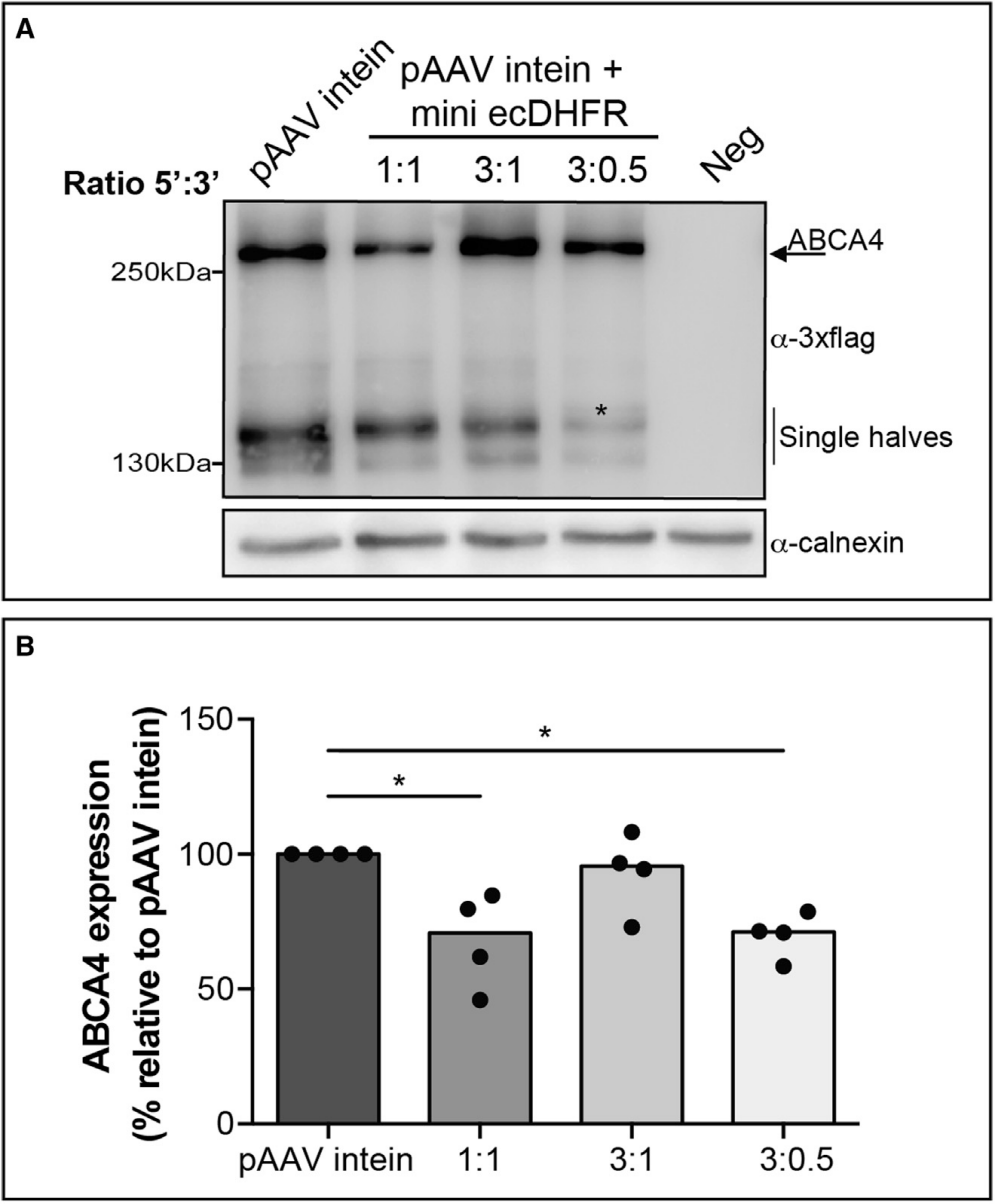
Increasing the amount of N-ABCA4 results in high full-length protein expression *in vitro* (A) Western blot (WB) analysis of lysates from HEK293 cells transfected with different ratios of the 5′ and 3′ AAV intein ABCA4-mini ecDHFR plasmids. The arrow and the asterisk indicate the bands corresponding to the full-length ABCA4 protein (ABCA4) and the N-ABCA4, respectively. Neg: untransfected cells. The WB is representative of N = 4 independent experiments. (B) Quantification of ABCA4 bands' intensity from AAV intein plasmids transfected at different ratios. The Kruskal-Wallis test followed by Dunn's test was used to identify statistically significant differences between groups; ∗p < 0.05. Results are represented as a single measurement (dot) and as median for each group of treatment (column).

The suboptimal full-length ABCA4 reconstitution observed *in vitro* when using the degron (Figure 3B) is presumably due to the lower levels of N- than C-ABCA4 half-polypeptide (Figure S6) available for PTS. These lower levels are the result of mini ecDHFR-mediated degradation of the N-polypeptide before PTS. In an effort to optimize the efficiency of AAV-ABCA4 intein + mini ecDHFR, we investigated whether increasing the levels of N- relative to C-ABCA4 improves full-length protein reconstitution as well as reducing the leftover of C-polypeptide after PTS has occurred. Thus, HEK293 cells were co-transfected with plasmids encoding both the 5′ and 3′ ABCA4 halves combined at different ratios. We found that the best 5′:3′ ratio was 3:1 (Figure 4), further increases of this ratio resulted in excessive amounts of 5′ half plasmid and the resultant N-polypeptide (Figure 4A). The 3:1 ratio gave similar or higher levels of full-length ABCA4 than those obtained in the absence of the degron (Figure 4).

We then evaluated the ABCA4 protein reconstitution mediated by AAV-ABCA4 intein + mini ecDHFR at the 3:1 ratio in the retinas of wild-type mice and pigs, the latter of which are similar in size and structure to human retinas^22^ and thus are excellent pre-clinical large animal models.

**Figure 5:**
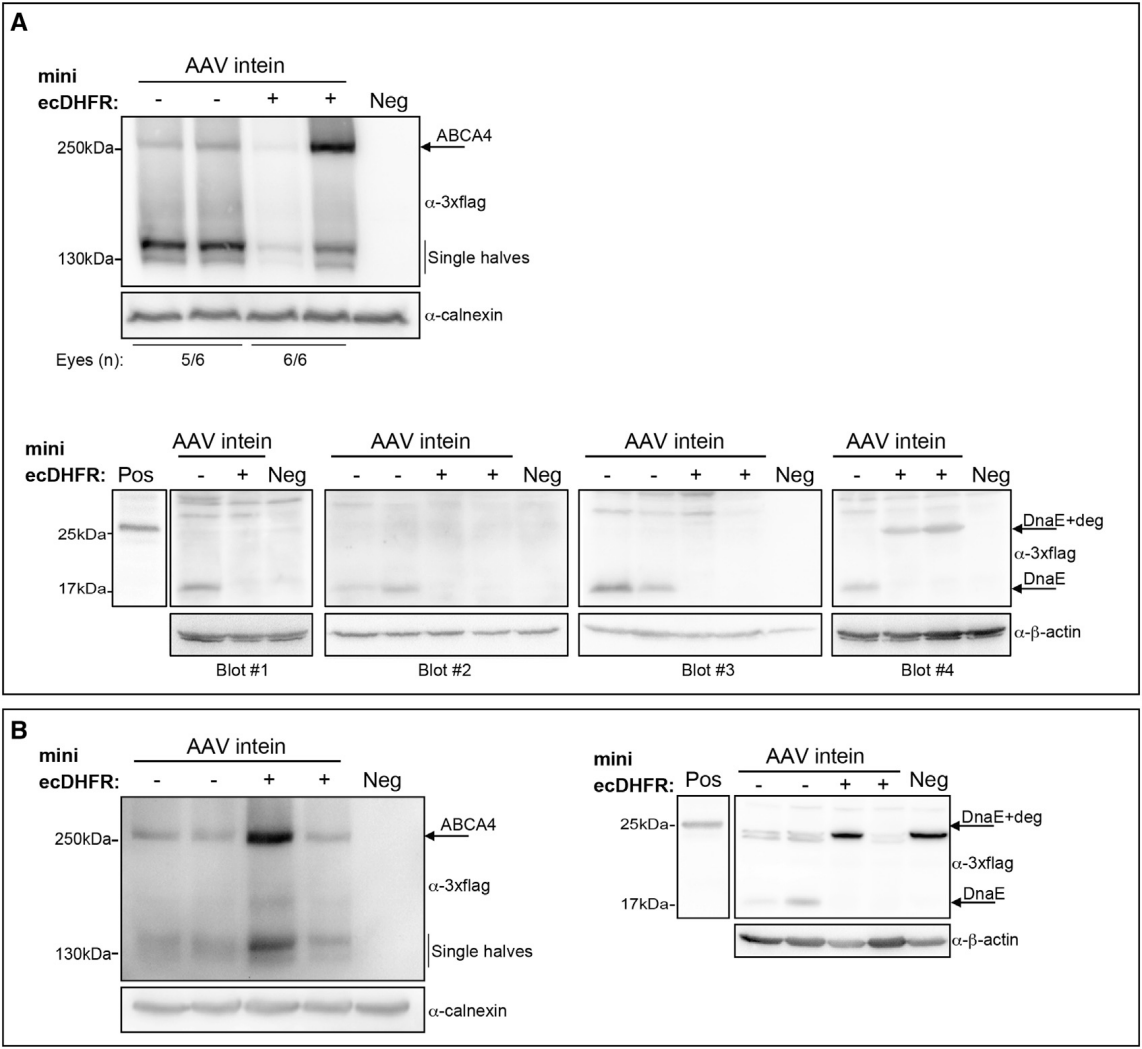
Efficient ABCA4 expression in the mouse and pig retina using three times more 5′ than 3′ vector Western blot analysis of retinal lysates from either wild-type mice (A) or Large White pigs (B) injected with AAV8-GRK1-ABCA4 intein vectors including (+) or not including (−) the mini ecDHFR degron. The arrows indicate the bands corresponding to full-length ABCA4 protein (ABCA4, [A] upper and [B] left panel) while excised inteins including (DnaE + deg) or not including (DnaE) the mini ecDHFR degron are shown in the lower (A) and right panel (B). Neg: PBS-injected eye. Pos: cell lysate of HEK293 cells transfected with ABCA4 intein-ecDHFR plasmids and treated with a proteasome inhibitor (MG132) to inhibit protein degradation since mini ecDHFR has lost trimethoprim sensitivity. The number of eyes showing full-length ABCA4 ([A] upper) out of the total eyes analyzed is indicated below each lane; excised inteins (DnaE and DnaE + deg) or absence of them for each eye are shown ([A] lower). A total number of two pig eyes were analyzed for each group (B).

Both wild-type mice and Large White pig eyes were subretinally injected with AAV8-GRK1-ABCA4 intein either with mini ecDHFR or without. One month later, we found that about 50% of the eyes injected with the AAV intein + degron at the 3:1 ratio outperformed those injected with the AAV intein 1:1 ratio in both mice (Figure 5A, upper) and pigs (Figure 5B) and showed robust ABCA4 expression. However, in two of seven mouse eyes injected with the 3:1 ratio, we found detectable levels of excised inteins (Figure 5A, lower). This suggests that the increased levels of PTS and excised intein obtained with the 3:1 ratio might saturate the cellular degradation machinery.

### Subretinal administration of AAV intein-mini ecDHFR reduces lipofuscin accumulation in a mouse model of STGD1

The accumulation of lipofuscin in the RPE which results from *Abca4* deficiency is the main hallmark in the STGD1 mouse model.^20^ Here, we assess the reduction of lipofuscin granules upon subretinal administration of AAV intein-mini ecDHFR vectors (at 3:1 ratio) in *Abca4*^*−/−*^ mice.

**Figure 6:**
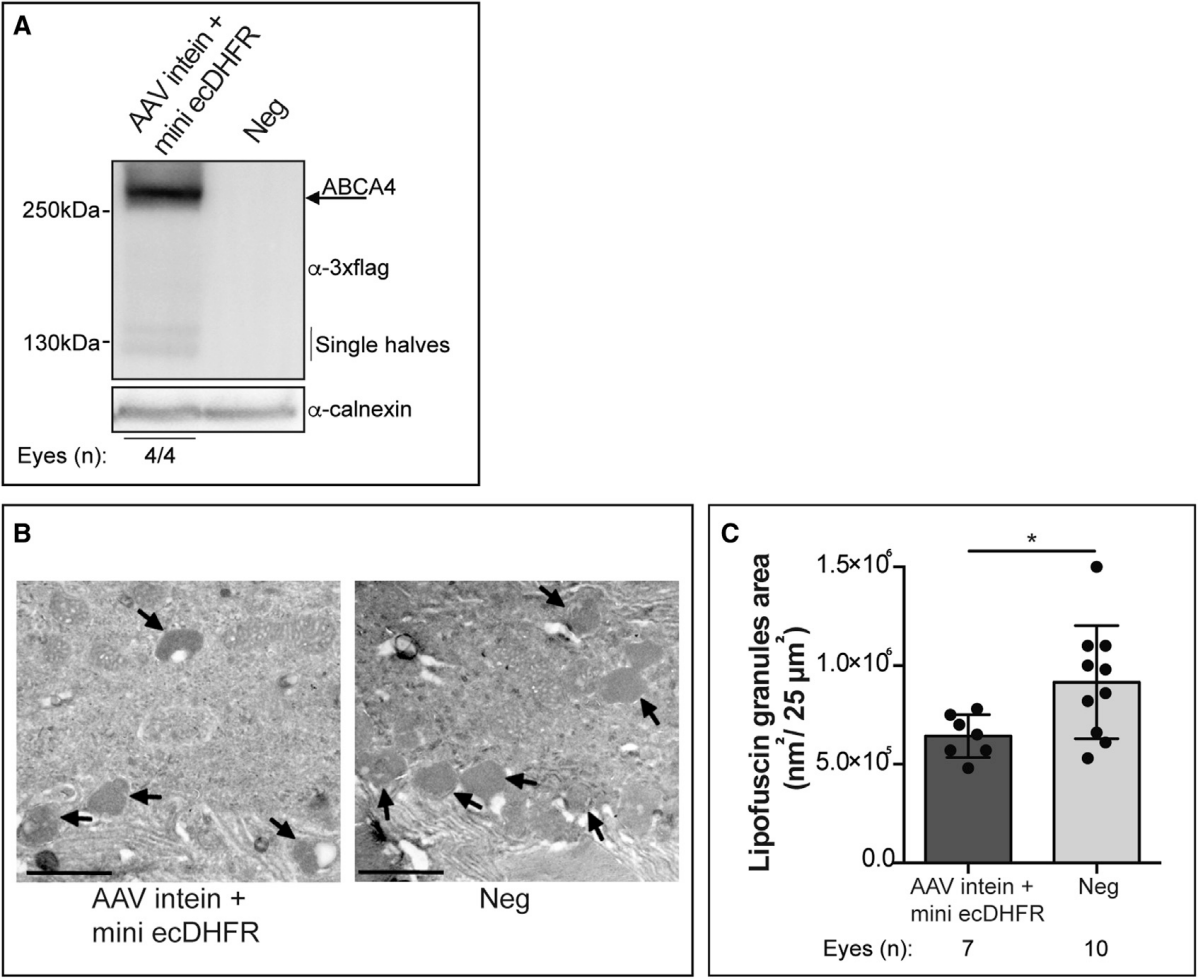
Subretinal delivery of AAV intein ABCA4 mini ecDHFR vectors results in significant reductions of lipofuscin granules in STGD1 mice Western blot analysis (A), representative images (B), and quantification (C) of the mean area occupied by lipofuscin in the RPE of either albino *Abca4*^*−/−*^ sham-injected (Neg: PBS-injected eye) or *Abca4*^*−/−*^ mice injected with AAV intein-mini ecDHFR vectors. (B) Representative lipofuscin granules are indicated in each image by arrows; scale bar, 1μm. (C) Statistical analysis was performed using the unpaired t test; ∗p < 0.05. Results are represented as a mean value for each mouse (dot) and as a mean ± SEM for each group of treatment (column).

One-month-old albino *Abca4*^*−/−*^ mice were injected subretinally with AAV8-GRK1-ABCA4 intein vectors and full-length ABCA4 expression was observed (Figure 6A). Three months later, the eyes were harvested in dark conditions, processed, and analyzed by electron microscopy.

We found that lipofuscin accumulation in the RPE of *Abca4*^*−/−*^ mice was significantly (p = 0.018) reduced in AAV eyes compared with sham-treated eyes (Figure 6). This is a partial reduction since lipofuscin accumulation in age-matched heterozygous *Abca4*^*−/+*^ is 4.1 × 10^5^ ± 2.28 × 10^4^ nm^2^/25 μm^2^.

### AAV intein-mini ecDHFR vectors are safe in the mouse retina

**Figure 7:**
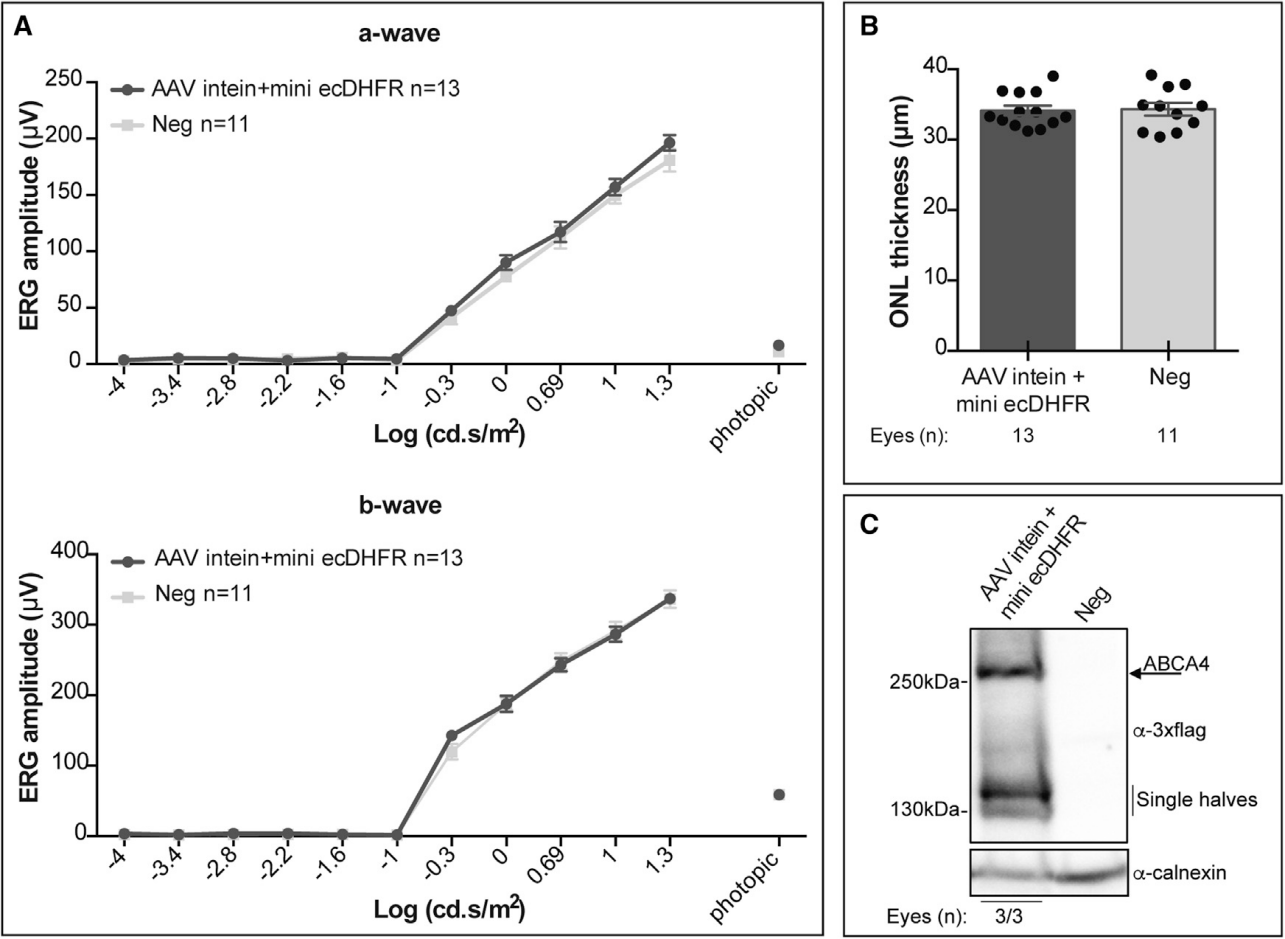
Subretinal administration of AAV intein + mini ecDHFR vectors is safe in mice up to 1 year postinjection Electroretinogram (ERG) mean a- and b-wave amplitudes (A), ONL (B), and ABCA4 protein expression (C) in C57BL/6J mice at 1 year post subretinal injection of AAV intein vectors (AAV intein + mini ecDHFR) or formulation buffer (Neg). Statistical analysis for each amplitude (A) was performed as detailed in the statistical analysis paragraph. The unpaired t test was used in (B); results are represented as a mean value for each mouse (dot) and as a mean ± SEM for each group of treatment (column).

To investigate the safety of the AAV-ABCA4 intein-mini ecDHFR vectors, wild-type C57BL/6J mice were injected subretinally with 9 × 10^9^ + 3 × 10^9^ genome copies (GC) per eye of 5′and 3′ vectors, respectively. Retinal electrical activity and thickness of the outer nuclear layer (ONL) were measured at 1 year after subretinal injection upon confirmation of ABCA4 protein expression (Figure 7). The amplitude of both a- and b-waves at any luminance was comparable between eyes injected with AAV intein + mini ecDHFR vectors and control eyes injected with formulation buffer (Figure 7A). Consistent with this, the two groups of eyes showed similar ONL thickness (Figure 7B). In addition, no histopathological changes were observed in the treated compared with negative control eyes.

## Discussion

In several instances, AAV intein represents the most efficient system to deliver large genes via AAV^9^^,^^10^, thus overcoming one of their major limitations which derives from their small size and associated cargo capacity.^6^ To improve the safety of this promising platform, in this study we investigated strategies that selectively mediate degradation of non-mammalian inteins that remain after PTS and full-length protein reconstitution.

Herein we showed that ecDHFR placed at the 3′ end of the N-intein mediates selective N- + C-intein degradation, while none of the other degradation signals tested significantly reduce the amount of the excised inteins and/or reduce the efficiency of *trans-*splicing and full-length ABCA4 reconstitution.

Although ecDHFR is a bacterially derived peptide, we reasoned that its short-lived expression is preferable to the persistence of the excised inteins. On the other hand, degron-triggered intein proteasomal degradation might generate intein peptides that are then presented through the major histocompatibility class I to CD8+ T cells. However, this may be less of a concern in photoreceptors, which are not known to act as antigen-presenting cells.^23^ However, since photoreceptor outer segments are normally phagocytized by retinal pigmented epithelium, which can act as antigen-presenting cell ^24^ and trigger an immune response, further investigations in this direction will be required to rule out this potential issue. However, several studies that use a combination of ecDHFR and TMP to control transgene expression in the retina^25^^,^^26^ and other tissues^21^^,^^27^ support the safety of this approach. In line with this, we found no adverse events and no changes to either retinal function or morphology in wild-type mice treated with AAV intein-ecDHFR vectors.

Unfortunately, ecDHFR is the largest among the degradation signals tested, which limits its applicability in the context of AAV. For this reason, we developed a shorter version (mini ecDHFR) that we show retains the original protein degradation activity without significantly impacting on levels of full-length ABCA4 reconstituted. This was obtained by preserving the amino acidic residues involved in protein degradation, while deleting those involved in sensitivity to TMP (Figure S3), which is not a relevant feature for the use of ecDHFR that we are proposing and will, in fact, eliminate potential interference by this widely used antibiotic.

ecDHFR retains a strong degradation activity when placed at the C terminus of a protein (Figure S6). This reduces the levels of N-ABCA4 polypeptide available for PTS, which we show can be rescued by increasing the dose of AAV intein 5′ vector delivered. Alternatively, to avoid the partial N-ABCA4 degradation, a proteasome inhibitor could be administered for a short time window to prevent ecDHFR activity before PTS has occurred, and then withdrawn once the full-length protein is reconstituted. In addition, the ecDHFR degradation activity could in theory be exerted on the single released N-intein to which it is linked: as a consequence, we cannot exclude the presence of residual C-intein, which cannot be detected because it is untagged.

Conversely, ecDHFR partial degradation activity on a single half-polypeptide can be used to reduce its levels when it exceeds those of the cognate half. This is the case for the AAV intein EGFP set in which the N- is more abundant than the C-polypeptide leaving excess N-polypeptide after PTS has occurred (Figure S2). In this case, when we fused ecDHFR to the N-EGFP polypeptide, its levels were reduced and matched those of the C-EGFP polypeptide counterpart resulting in similar amounts of full-length EGFP reconstitution without any residual N-polypeptide. Therefore, the partial degradation activity that ecDHFR exerts on single polypeptides expressed from AAV intein can be used to our advantage to modulate polypeptide levels to match each other, when applicable.

We show that subretinal delivery of AAV intein-mini ecDHFR vectors is safe in wild-type mice at the same doses that were effective in *Abca4*^*−/−*^ animals. These doses, when extrapolated to the human retina, which has an overall area about 70- to 100-fold larger than the mouse,^28^^,^^29^ fall within doses that are being safely evaluated in patients. Formal nonclinical safety/toxicity studies, as well as a further characterization of ecDHFR-mediated intein degradation, including a thorough evaluation of the fate of both the single N- and C-inteins after PTS, is required before future translation of AAV intein-mini ecDHFR from bench to bedside.

In conclusion, we show that inclusion of ecDHFR in AAV intein vectors results in efficient protein reconstitution in the absence of significant intein persistence. The safe and effective delivery of these optimized vectors to the retina of a mouse model of STGD1 supports their further translation for gene therapy of STGD1 as well as of other inherited retinal diseases.

## Materials and methods

### Generation of AAV vector plasmids

The plasmids used for AAV vector production derived from either the pZac^30^ or the pTIGEM plasmid that contains both the ITRs of AAV serotype 2 and either the ampicillin or the kanamycin resistance, respectively. The EGFP and ABCA4 intein plasmids used in this study were the same plasmids (with the same expression cassette) originally described in Tornabene et al.^9^ Specifically, we chose the most efficient set of ABCA4 intein plasmids (set 1) tested in the previous study.^9^ Briefly, The ABCA4 protein was split in the large CD1 cytoplasmic domain before amino acid (aa) C1150, while EGFP was split at aa C71. To detect either ABCA4 or EGFP full-length protein, a triple flag (3x-flag) tag was added at the C-terminal end of the C-polypeptide. Inteins included in both plasmids were those derived from DnaE of *Nostoc punctiforme.*^31^^,^^32^

Plasmids encoding EGFP included the bovine growth hormone polyadenylation signal and the ubiquitous cytomegalovirus (CMV^30^) promoter.

Plasmids encoding ABCA4 included the simian virus 40 polyadenylation signal and either the short CMV^33^ or the photoreceptor-specific human G protein-coupled receptor kinase 1^34^ promoters for *in vitro* and *in vivo* studies, respectively.

All the degradation signals were synthesized and cloned by In-Fusion (Takara, Kusatsu, Japan) either at the 5′ or 3′ ends of the half coding sequence. The mini ecDHFR was mutagenized from the plasmid containing the 5′ ABCA4-ecDHFR by In-Fusion (Takara). To detect any intein-degron fusion proteins, a 3x-flag tag was added at the C-terminal end of the N-intein. A schematic representation of those plasmids can be found in Figure S1.

### AAV vector production and characterization

AAV vectors serotype 8 (AAV8) were produced by the TIGEM AAV Vector Core by triple transfection of HEK293 cells, as already described.^35^ For each viral preparation, physical titers (GC/mL) were determined by averaging the titer achieved by dot-blot analysis and by PCR quantification using TaqMan^35^ (Applied Biosystems, Carlsbad, CA).

### Culture and transfection of HEK293 cells

Human embryonic kidney 293 (HEK293) cells were maintained and transfected using the calcium phosphate method (1 μg of each plasmid/well in 6-well plate format), as already described.^36^ The maximum material transfected was 4 μg. The total amount of DNA transfected in each well was kept equal through the addition of a scramble plasmid where necessary. For the experiments in Figures 2E, S2, and S3, 24 h after transfection, cells were treated with 100 μM of TMP (T7883; Sigma-Aldrich St. Louis, MO); in Figures 3B, 5, S3, and S5, cells were treated with 20 μM of MG132 (474,787; Sigma-Aldrich) for 12 h before harvesting the cells.

### Western blot analysis

Samples (HEK293 cells and mouse and pig retinas) were lysed in RIPA buffer (supplemented with protease inhibitors and 1 mM phenylmethylsulfonyl) to extract proteins. After lysis, ABCA4 samples were denatured at 37°C for 15 min in 1x Laemmli sample buffer supplemented with 2M urea. EGFP and DnaE-intein samples were denatured at 99C for 5 min in 1x Laemmli sample buffer. Lysates were separated by either 12% (for EGFP and DnaE-intein samples) or 6% (for ABCA4 sample) SDS-PAGE. The antibodies used for immuno-blotting are as follows: anti-3xflag (1:2000, A8592; Sigma-Aldrich) to detect the EGFP, ABCA4, and DnaE-intein proteins; anti-β-actin (1:2000, NB600-501; Novus Biological LLC) to detect β-actin used as loading control for small protein size; anti-calnexin (1:2000, ADI-SPA-860; Enzo life sciences, Axxora LLC) to detect calnexin used as loading control for large protein size. The quantification of EGFP, ABCA4, and DnaE-intein bands detected by western blot was performed using ImageJ software (free download available at https://imagej.nih.gov/ij/download.html).

### Subretinal injection of AAV vectors in mice and pigs

Animals were kept in a pathogen-free like facility (TIGEM animal house, Pozzuoli Italy) and maintained under a 12-h light/dark cycle. Animals were raised in accordance with the Institutional Animal Care and Use Committee guidelines for the care and use of animals in research.

Studies in animals were carried out in accordance with both the Association for Research in Vision and Ophthalmology Statement for the Use of Animals in Ophthalmic and Vision Research and with the Italian Ministry of Health regulation for animal procedures (Ministry of Health authorization number: 147/2015-PR). C57BL/6J mice were purchased from Envigo Italy SRL (Udine, Italy). Albino *Abca4*^*−/−*^ mice were generated through successive crosses and backcrosses with BALB/c (homozygous for Rpe65 Leu450) and maintained inbred.

The Large White female pigs (imported from Azienda Agricola Pasotti, Imola, Italy) used in this study were registered as purebred in the LWHerd Book of the Italian National Pig Breeders' Association and were housed at the Centro di Biotecnologie A.O.R.N Antonio Cardarelli (Naples, Italy) and maintained under 12-h light/dark cycle. Surgery was performed under general anesthesia, and all efforts were made to minimize animal suffering. Mice were anesthetized with an intraperitoneal injection of 10 μL/gr of body weight of ketamine (10 mg/Kg) combined with medetomidine (1 mg/Kg). AAV8 vectors were subsequently delivered subretinally via a trans-scleral trans-choroidal subretinal injection, as described by Liang et al.^37^ Eyes were injected with 1μL of vector solution, dose of each vector/eye: 3 × 10^9^ GC.

Subretinal delivery of AAV8 vectors to the pig retina was performed as previously described.^38^ Pigs were anesthetized with an intramuscular injection of Zoletil (0.5 mL/kg), ketamine (10 mg/kg), and propofol (6 mg/kg). Eyes were injected with two blebs of 100 μL of AAV8 vector solution, dose of each vector/eye: 1 × 10^11^ GC. AAV8 vectors were used because of their high efficiency of photoreceptor transduction.^39^

### Transmission electron microscopy analysis

To evaluate lipofuscin accumulation in the RPE of the STGD1 mouse model, *Abca4*^*−/−*^ mice were injected with AAV intein-mini ecDHFR vectors. Three months postinjection, mice were dark-adapted for 8 h and scarified under dim red light. Eyes were fixed, processed, and analyzed as previously described.^9^

### Histology and histopathology

To evaluate retinal ONL thickness on retinal histological sections, C57BL/6J mice eyes were fixed overnight in Davidson fixative (deionized water, 10% acetic acid, 20% formalin, 35% ethanol), dehydrated by serial passages in ethanol, and then embedded in paraffin blocks. Ten-micrometer-thick microsections were cut along the horizontal meridian, progressively distributed on slides, and stained with Harris Hematoxylin and Eosin (Sigma-Aldrich). Then, the sections were analyzed under the microscope (Leica Microsystems GmbH DM5000, Wetzlar, Germany) and acquired at ×20 magnification. For each eye, three images from the temporal injected side of different depth (ventral, central, and dorsal side) representative of the whole eye were used for both histology and histopathology analyses. For the histology, three measurements of the ONL thickness were taken for each image using the “freehand line” tool of the ImageJ software. The histopathology analysis was carried out by a certified-pathologist at Cogentech S.c.a.r.l. Both analyses were done blind to the treatment group.

### Electroretinogram recordings

Functional analyses in mice were performed as detailed in^36^

### Statistical analysis

Data are presented either as median (for nonparametric data) or mean ± SEM, which have been calculated using the number (n) of independent *in vitro* experiments or retinas (not replicate measurements of the same sample). Statistical p values <0.05 were considered significant. The normality assumption of the homogeneity of variances were verified by the Shapiro-Wilk (p value >0.05) and Levene's tests (p value >0.05), respectively. Data were analyzed by (1) Student's t test, (2) Wilcoxon rank-sum test (nonparametric test), (3) Kruskal-Wallis rank-sum test (nonparametric test), and (4) Welch t test (unequal variances t test). Pairwise comparison between group levels with correction of multiple testing were performed to determine whether the difference between specific pairs of groups is statistically significant. Specific statistical values were made as follows:

- Figure 3B. The Kruskal-Wallis test followed by the Dunn's test were used to identify statistically significant differences between groups. The Kruskal-Wallis test p value is 0.092.
- Figure 4B. The Kruskal-Wallis test followed by the Dunn's test were used to identify statistically significant differences between groups. The Kruskal-Wallis test p value is 0.017. Post hoc p values are as follows: pAAV intein versus 1:1 = 0.040; pAAV intein versus 3:1 = 0.492; pAAV intein versus 3:0.5 = 0.040 1:1 versus 3:1 = 0.149; 1:1 versus 3:0.5 = 0.822; 3:1 versus 3:0.5 = 0.122.
- Figure 6C. The unpaired t test was used to compare the mean of two independent groups; the p value is 0.018.
- Figure 7. (A) Different statistical tests were used to compare AAV intein + mini ecDHFR and Neg groups at different lux (see Table 2). (B) The unpaired t test was used to compare two independent groups; the p value is 0.870.

**Table 2:** Statistical analysis used in Figure 7A

Lux [Log (cd.s/m^2^)]	a-wave		b-wave	
	p value	Method	p value	Method
−0.3	0.443	Unpaired t test	0.970	Unpaired t test
−1	0.443	Unpaired t test	0.970	Unpaired t test
−1.6	0.443	Unpaired t test	0.970	Unpaired t test
−2.1	0.136	Unpaired t test	0.970	Unpaired t test
−2.8	0.727	Unpaired t test	0.742	Unpaired t test
−3.4	0.478	Unpaired t test	0.764	Unpaired t test
−4	0.211	Unpaired t test	0.953	Unpaired t test
0	0.443	Unpaired t test	0.234	Unpaired t test
0.7	0.425	Unpaired t test	0.234	Unpaired t test
1	0.531	Unpaired t test	0.234	Unpaired t test
1.3	0.531	Unpaired t test	0.234	Unpaired t test
2.4	0.531	Welch t test	0.999	Unpaired t test
- Figure S4B. The Wilcoxon rank-sum test was used to compare EGFP amounts from pAAV EGFP-intein plasmids with or without the mini ecDHFR. The Wilcoxon rank-sum test p value is 0.640. The Kruskal-Wallis test followed by the Dunn's test were used to compare intein amounts from pAAV EGFP-intein plasmids with or without the mini ecDHFR. The Kruskal-Wallis test p value is 0.024. Post hoc p values are as follows: EGFP versus DnaE = 0.170; EGFP versus DnaE + mini ecDHFR = 0.170; DnaE versus DnaE + mini ecDHFR = 0.018.

## Supplemental information

Document S1. Figures S1–S6

Document S2. Article plus supplemental information
