# Supplementary material for: Inclusion of a degron reduces levelsof undesired inteins after AAV-mediated proteintrans-splicing in the retina
Source: Mol Ther Methods Clin Dev. 2021 Oct 19;23:448–59. doi: 10.1016/j.omtm.2021.10.004 (PMC8571531; doi:10.1016/j.omtm.2021.10.004)
Supplement: Document S1. Figures S1–S6 [file mmc1.pdf]

OMTM, Volume *23*

Supplemental information

Inclusion of a degron reduces levels of undesired inteins after AAV-mediated protein *trans*-splicing in the retina

Patrizia Tornabene, Ivana Trapani, Miriam Centrulo, Elena Marrocco, Renato Minopoli, Mariangela Lupo, Carolina Iodice, Carlo Gesualdo, Francesca Simonelli, Enrico M. Surace, and Alberto Auricchio

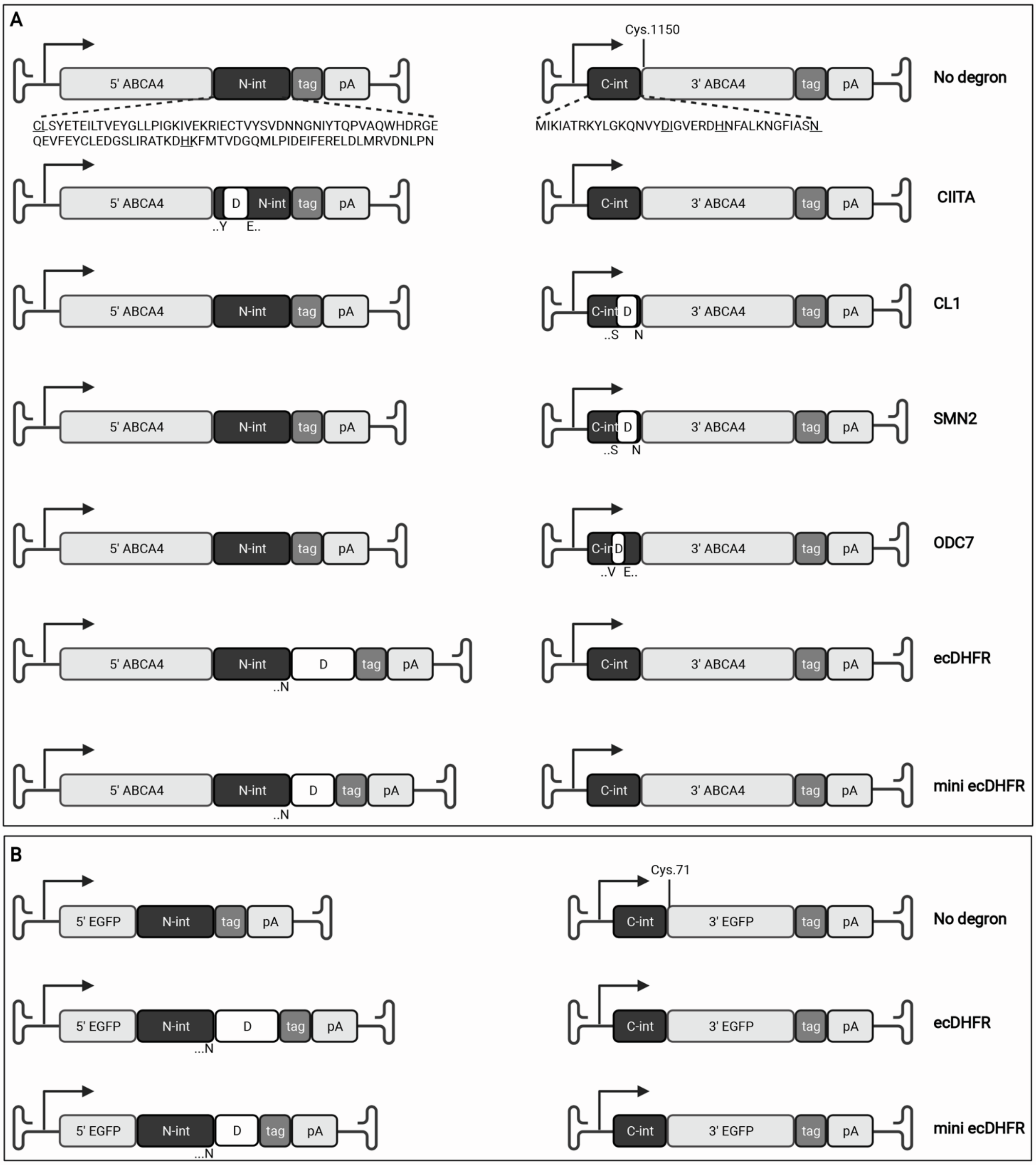

Figure S1. Schematic representation of the AAV intein-degron vectors.

Schematic representation of both ABCA4 (**A**) and EGFP (**B**) intein vectors including or not the degradation signal (reported on the right). The amino acid sequence (with catalytic residues underlined) for both N- and C-inteins (**A**) as well as the splitting point (for both ABCA4 and EGFP) are shown in the original constructs without degradation signal. Amino acids flanking each degradation signal are highlighted. The arrows indicate either the short (for ABCA4) or the full-

length cytomegalovirus (for EGFP) promoter. D: degron; tag: 3xflag tag; pA: either simian virus 40 (for ABCA4) or bovine growth hormone (for EGFP) polyadenylation signal; inverted terminal repeats are depicted at the two ends of each vector.

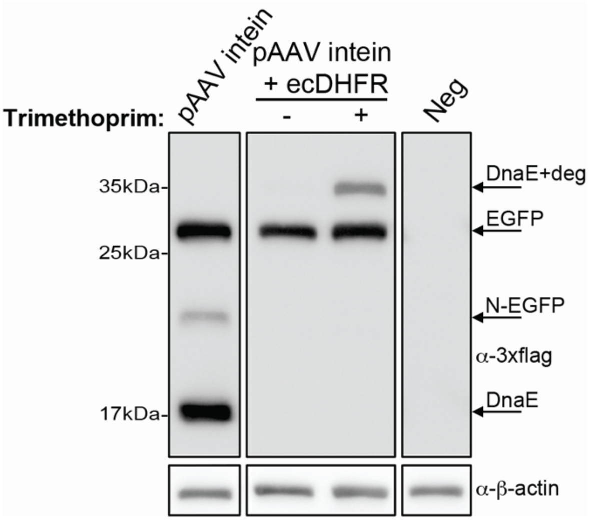

Figure S2. E. coli DHFR induces selective intein degradation in cells transfected with AAV-EGFP-intein plasmids.

Western blot (WB) analysis of lysates from HEK293 cells transfected with AAV intein-ecDHFR plasmids containing (+) or not containing (-) the stabilizer Trimethoprim. The arrows indicate the bands corresponding to full-length EGFP protein (EGFP), the N-terminal of EGFP (N-EGFP), and the excised inteins including (DnaE+deg) or not including (DnaE) the ecDHFR degron. Neg: untransfected cells. The WB is representative of N=3 independent experiments.

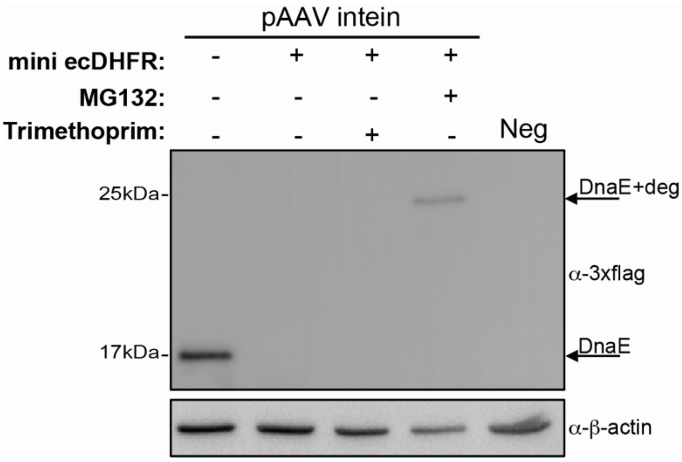

Figure S3. Trimethoprim does not inhibit mini ecDHFR-mediated intein degradation.

Western blot analysis of lysates from HEK293 cells transfected with AAV ABCA4 intein plasmids including (+) or not including (-) the mini ecDHFR degron, the proteasome inhibitor MG132, and the stabilizer Trimethoprim. The arrows indicate the bands corresponding to excised inteins including (DnaE+deg) or not including (DnaE) the mini ecDHFR degron. Neg: untransfected cells.

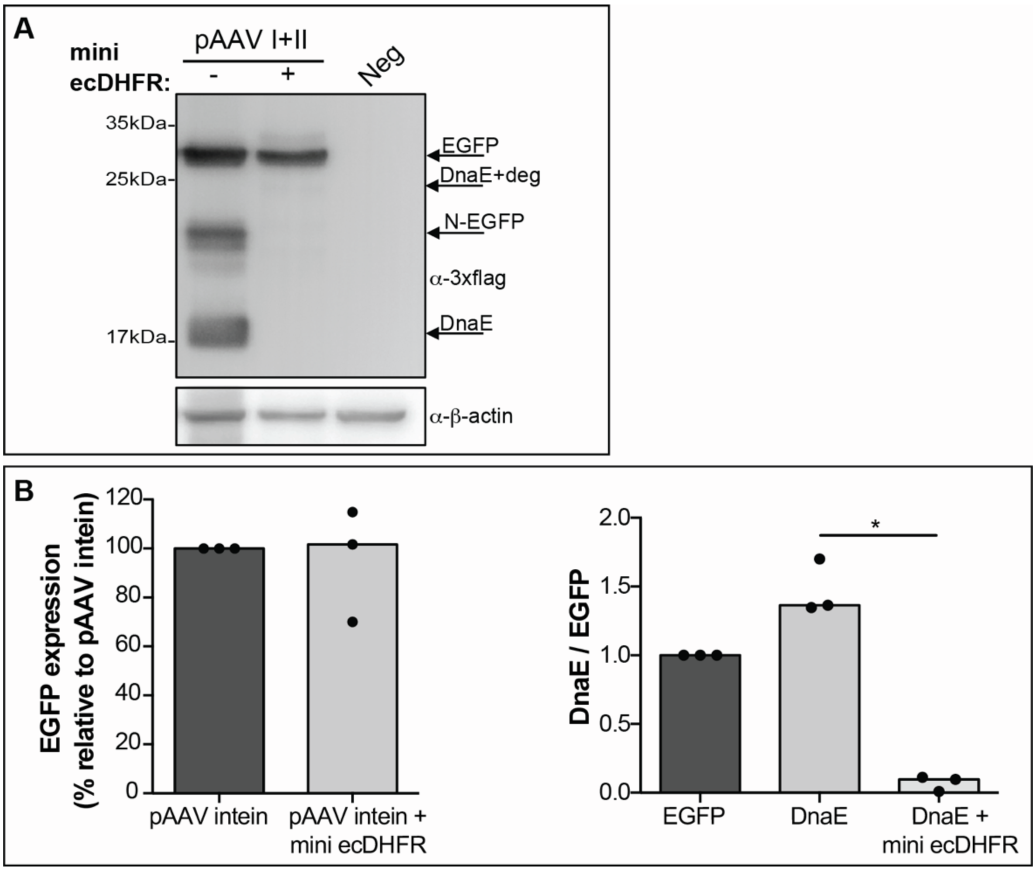

Figure S4. Mini ecDHFR induces selective intein degradation while preserving similar EGFP expression as cells co-transfected with EGFP intein AAV vectors.

(**A**) Western blot (WB) analysis of lysates from HEK293 cells transfected with AAV intein plasmids either including (+) or not including (-) the mini ecDHFR degron. The arrows indicate the full-length EGFP protein (EGFP), the N-terminal of EGFP (N-EGFP), and the excised inteins including (DnaE+deg) or not including (DnaE) the mini ecDHFR degron. Neg: untransfected cells. The WB is representative of N=3 independent experiments. (**B**) Quantification of EGFP expression from pAAV intein + mini ecDHFR relative to pAAV intein is shown in the upper graph. Quantification of the excised inteins bands including (DnaE+deg) or not including (DnaE) the mini ecDHFR degron vs full-length EGFP is shown in lower graph. Statistical comparisons were made using the Wilcoxon test p=0,640 (EGFP expression) and the Kruskal-Wallis test followed by Dunn’s test to identify statistically significant differences between groups (DnaE expression); *= $p<0.05$. Results are represented as a single measurement (dot) and as median for each group of treatment (column).

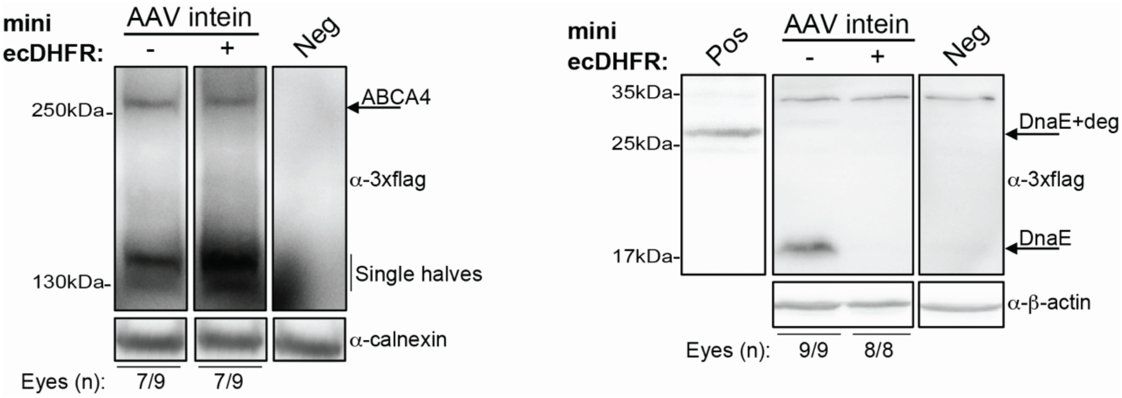

Figure S5. Mini ecDHFR induces selective intein degradation in mouse photoreceptors.

Western blot analysis of retinal lysates from wild-type mice injected with AAV-GRK1-ABCA4 intein vectors including (+) or not including (-) the mini ecDHFR. The arrows indicate the bands corresponding to full-length ABCA4 protein (ABCA4, left panel) while excised inteins, including (DnaE+deg) or not including (DnaE) the mini ecDHFR degron, are shown in the right panel. Neg: PBS-injected eye. Pos: cell lysate of HEK293 cells transfected with ABCA4 intein-mini ecDHFR plasmids and treated with a proteasome inhibitor (MG132) to inhibit protein degradation since the mini ecDHFR has lost Trimethoprim sensitivity. The number of eyes showing either full-length ABCA4 (left panel); excised inteins (DnaE) or absence of them (right panel) out of the total eyes analyzed is indicated below each lane.

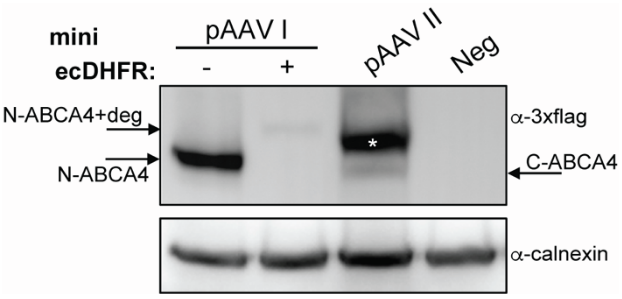

Figure S6. Mini ecDHFR retains degradation activity at the C-terminal end of the N-polypeptide.

Western blot (WB) analysis of lysates from HEK293 cells transfected with plasmids encoding for the 5' (pAAV I) and 3' (pAAV II) halves of the protein either including (+) or not including (-) the mini ecDHFR degron. The arrows indicate the N-terminal of ABCA4 protein including (N-ABCA4+deg) or not including (N-ABCA4) the mini ecDHFR degron and the C-terminal of ABCA4 (C-ABCA4). *protein product with a potentially different post-translational modification. The WB is representative of N=3 independent experiments.
